# Carcinome épidermoïde verruqueux palpébrale: à propos d'un cas

**DOI:** 10.11604/pamj.2014.18.93.4248

**Published:** 2014-05-27

**Authors:** Nazih Tzili, Oubaida El Yamouni, Mahfoud El Khaoua, Zakaria Mellal, Sanae Sefiani, Amina Berraho

**Affiliations:** 1 Service d'Ophtalmologie B, Hôpital des Spécialités, CHU, Rabat Maroc; 2 Service d'Anatomie Pathologique, Hôpital des Spécialités, CHU, Rabat Maroc

**Keywords:** Carcinome épidérmoide, carcinome verruqueux, tumeur palpébrale, virus du papillome humain, squamous carcinoma, verrucous carcinoma, eyelid tumor, human papillomavirus

## Abstract

Le carcinome épidermoïde verruqueux est une forme rare du carcinome épidermoïde et se caractérise par une malignité essentiellement locale. La localisation palpébrale est exceptionnelle. Nous rapportons le cas d'une patiente de 70 ans, suivi en consultation pour tumeur palpébrale volumineuse simulant un kératoacanthome de l’œil droit. La biopsie révèle un carcinome épidermoïde verruqueux. Une exérèse chirurgicale de la totalité de la tumeur avec reconstruction de la paupière ont été réalisées. On n'a pas noté de récidive pendant les 6 mois suivant le traitement chirurgical. Il s'agit du quatrième cas de carcinome épidermoide verruqueux à localisation palpébrale reporté dans la littérature.

## Introduction

Le carcinome épidermoïde verruqueux est une forme rare du carcinome épidermoïde. Il se caractérise par une malignité essentiellement locale. Sa localisation palpébrale est exceptionnelle.

## Patient et observation

**Figure 1:**
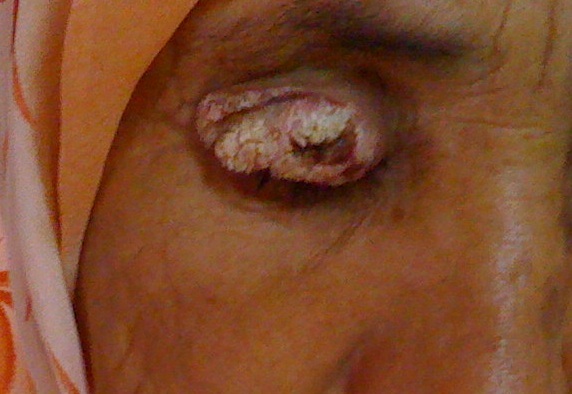
Une lésion tumorale blanchâtre, kératosique et exophytique occupant toute la paupière supérieure droite mesurant 4 x 2 cm

**Figure 2:**
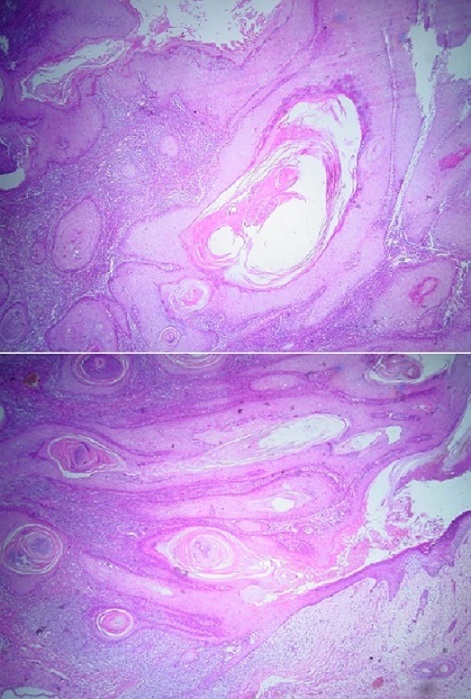
Une prolifération carcinomateuse papillomateuse faite de papilles bordées de cellules malpighiennes aux noyaux augmentés de taille, hyper chromatiques et fortement nucléolés. Un cytoplasme éosinophile. Absence d’éléments invasifs

**Figure 3:**
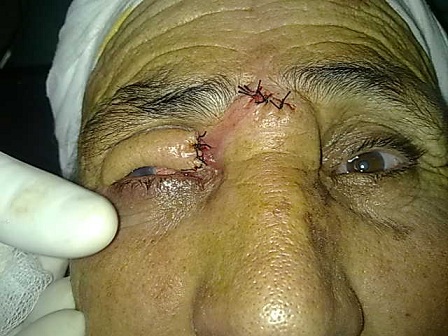
Photo de la patiente après reconfection du lambeau médio frontale

Patiente de 70 ans, ayant comme antécédent un traumatisme oculaire droit depuis l'enfance. L'examen ophtalmologique du coté droit retrouve: une acuité visuelle réduite aux mouvements des doigts avec une dystrophie de cornée homolatérale et une lésion tumorale blanchâtre, kératosique et exophytique occupant toute la paupière supérieure; ferme et bien limitée, faisant penser à un kératoacanthome, dont les mesures sont: 4 x 2 cm (Figure 1) évoluant depuis 3 ans. Du coté gauche, l'examen ophtalmologique est normal mis à part une cataracte. L'examen général et le bilan préopératoire sont normaux. L'examen histologique d'une biopsie à cheval sur la zone de peau saine et la lésion révèle un carcinome épidermoïde verruqueux (Figure 2). Le traitement consiste en une exérèse chirurgicale de la totalité de la tumeur avec reconstruction de la paupière supérieure droite par un lambeau médio-frontal (Figure 3). Les marges de l'exérèse sont saines. Une recherche de virus HPV par immunomarquage (ventana BenchMark Ultra) est négative. On note aucune récidive ni de métastase pendant les 3 ans suivant le traitement chirurgical.

## Discussion

Le carcinome épidermoïde verruqueux est un carcinome bien différencié de bas grade de malignité. Sa description a été faite par Ackermann en 1948 définissant ainsi ses critères cliniques et histologiques [1]. Le site de prédilection de ce type de carcinome est la peau, l’æsophage, la cavité buccale, le larynx, la face plantaire et les parties génitales [1]. La localisation palpébrale est exceptionnelle. Dans la littérature, trois cas de carcinomes épidermoïdes verruqueux à localisation palpébrale ont été rapportés [2–4] dont le premier cas a été publié en 1983. Comme la lésion est souvent indolente ayant la forme de chou-fleur, elle simule une grande verrue ou un Kératoacanthome qui est une tumeur épithéliale bénigne d’évolution aiguë dont seule la biopsie à cheval sur la zone de peau saine et la lésion peut permettre le diagnostic différentiel [5].

Concernant la pathogénicité du carcinome verruqueux, plusieurs facteurs de risque ont été évoqués dans l'implication du développement de ce type de cancer notamment le rôle du virus du papillome humain (VPH) et la sur-expression de l′oncogène p53 qui semblent être des facteurs étiologiques importants [6]. Dans notre cas, la recherche du (VPH) est négative.

L'exérèse totale avec des marges de sécurité constitue le meilleur traitement et sera indispensable en cas de doute clinique et histologique. Le pronostic du carcinome verruqueux est souvent favorable en raison de l′absence de métastases à distance. Néanmoins, il est localement agressif [7] nécessitant une chirurgie reconstructive à grande échelle en cas de retard de prise en charge.

## Conclusion

Le carcinome épidermoïde verruqueux est une tumeur à croissance lente et localement agressive dont le traitement de choix est l'exérèse chirurgicale en marges saines. Malgré sa localisation palpébrale exceptionnelle, il faut y penser devant toute lésion ressemblant à une grande verrue ou un Kératoacanthome afin d’éviter le retard thérapeutique.
